# Proteomics Analysis of Colostrum Samples from Sows Housed under Different Conditions

**DOI:** 10.3390/ani10020355

**Published:** 2020-02-22

**Authors:** Guoan Yin, Lei Wang, Xiaoyu Zhao, Langchao Yu, Dapeng Huang

**Affiliations:** 1 College of Animal Science and Veterinary Medicine, Heilongjiang Bayi Agricultural University, Daqing 163319, China; byndw5@163.com (L.W.); wzhaoxiaoyu@163.com (X.Z.); qaxylc@163.com (L.Y.); 2 Heilongjiang Key Laboratory of Efficient Utilization of Feed Resources and Nutrition Manipulation in Cold Region, Daqing 163319, China

**Keywords:** sow, colostrum, housing system, proteomics, signaling pathway

## Abstract

**Simple Summary:**

Milk quality in sows is affected by various factors, including the living environment. However, changes in protein-expression profiles in the milk of sows housed under different conditions are still unclear. Accordingly, in this study, we performed proteomics analysis of colostrum from sows housed under different conditions. Our results showed that housing conditions affected protein expression, and the effects of housing conditions were observed in both pregnancy and lactation. In comparison, the differentially expressed proteins were related to the immune system and metabolic processes, particularly fat metabolism. These results revealed the physiological changes in sows under different housing conditions. Overall, these findings provided important insights into the care and management of sows to enhance milk quality.

**Abstract:**

This study investigated the proteomic characteristics of colostrum for sows housed under different conditions. Among 12 gilts, four were housed in a gestation-crate and farrowing-crate combined housing system (CC) as controls, four were housed in a gestation-pen and farrowing-pen combined housing system (PP), and four were housed in a gestation-pen and farrowing-crate combined housing system (PC). Differentially expressed proteins in the colostrum (PP versus CC, and PC versus CC) were screened by proteomics technology, and bioinformatics analysis was then performed. Results showed that 93 proteins were differentially expressed in PP versus CC, and that 126 proteins were differentially expressed in PC versus CC. The differentially expressed proteins in the PP versus CC comparison were mainly enriched in interleukin (IL)-17, transforming growth factor-β, and nuclear factor-κ B signaling pathways, and in metabolic pathways, including glutathione metabolism, peroxisome, and carbon metabolism. In contrast, differentially expressed proteins in the PC versus CC comparison were enriched in the IL-17 signaling pathway, cholesterol metabolism, and peroxisome proliferator-activated receptor signaling pathway. In conclusion, the housing environment appeared to affect the colostrum composition of sows by acting on their immune system and metabolic processes, particularly fat metabolism.

## 1. Introduction

Sow-milk quality directly affects the growth performance and survival rates of piglets, which are related to productivity per sow per year and economic benefits. The synthesis and secretion of sow milk is regulated by many factors, including genetic factors, nutrition, and the housing environment. Many in-depth studies have evaluated the effects of genotype [1], nutrition [2,3], and lactation stage [4,5] on sow-milk quality; however, the housing environment has not been studied in detail. In previous studies, factors such as heat stress caused by temperature changes, chronic stress due to a restrictive environment (e.g., crates) [6], and lack of nesting materials before farrowing [7] were shown to alter the physiological processes in sows, including changes in oxytocin and prolactin secretion. Together, these factors decrease milk production and alter milk composition in sows.

The digestive system, thermotaxic center, and immune system of newborn piglets are immature [8]. Additionally, the external environment is cold and contaminated with pathogens; therefore, piglets must consume a certain amount of colostrum in order to maintain body temperature and to resist harmful pathogens [9,10]. Colostrum is complex in cows, containing over 200 proteins [2]. Importantly, colostrum mainly contains immunologically active materials, including immunoglobulins (IgG and a small amount of IgA and IgM) [5,11] and lactoferrin [12]; high-abundance proteins, including β-lactoglobulin, α-lactalbumin, and serum albumin; and low-abundance proteins, including β2-microglobulin, orotic acid protein, and fatty-acid-binding protein [4,13]. Proteomics techniques have provided a new approach to study milk proteins and resulted in major progresses in milk-proteomics research [14]. Thus, the complex components of sow colostrum could be identified using proteomics technology, and results could have important implications for the care and management of sows.

Accordingly, in this study, we performed proteomics analysis of colostrum from sows housed under different conditions using gene-ontology (GO) annotations and Kyoto Encyclopedia of Genes and Genomes (KEGG) analysis. Our results demonstrated the effects of a housing environment on sow-milk performance, which could contribute to improving sow feeding and management strategies.

## 2. Materials and Methods

### 2.1. Animals, Treatments, Management, and Feeding

This experiment was reviewed and approved by the Animal Ethics Committee of College of Animal Science and Veterinary Medicine, Heilongjiang Bayi Agricultural University (no. 2018-04), and was carried out at a commercial pig farm located in Qiqihar, China. In total, 16 Yorkshire × Landrace gilts were selected after insemination. All gilts were healthy and without clinical lameness, and weighed 135 ± 5 kg at insemination. Pregnancy was confirmed in 12 of the 16 gilts 21 days after insemination. These 12 gilts were then randomly assigned to three types of housing systems (four sows each): a gestation-crate and farrowing-crate combined housing system (CC) as controls, a gestation-pen and farrowing-pen combined housing system (PP), and a gestation-pen and farrowing-crate combined housing system (PC). The gestation crates were commercial gestation stalls, measuring 2.1 × 0.6 m, and were not covered with straw. The gestation pens measured 3.2 × 3.2 m (four sows each), and the ground was covered with straw (100 mm depth). The farrowing pens measured 3.5 × 2.0 m, allowing sows to move freely, and the ground was covered with straw (100 mm depth). Finally, farrowing crates measured 2.1 × 1.8 m and were not covered with straw; a stall measuring 2.1 × 0.6 m was installed to limit sow activities. Details of all housing systems were described by Yin et al. [15]. Sows were housed in the same room containing gestation crates or pens from 21 days after insemination to 7 days before the expected delivery date. All sows were transferred from the pregnancy unit to the farrowing unit 7 days before the expected delivery date (107 days after insemination), and all farrowing crates or pens were installed at the same unit. All sows were fed the same nutritional standard (NRC, 2012); details are shown in Table 1. Other management standards were carried out according to uniform standards for commercial pig farms.

### 2.2. Colostrum Samples

All colostrum samples were collected from the anterior, middle, and posterior mammary glands of sows at 0, 6, and 12 h after farrowing each sow (the time was considered to start from the birth of the first piglet). Nine labeled centrifuges were used to collect the colostrum. Then, colostrum samples were mixed in balanced quantities. In total, 5–10 mL colostrum was sampled from each sow and collected into polypropylene plastic bottles. The collected milk samples were marked, transported at 4 °C, and then stored at −80 °C in a refrigerator.

### 2.3. Isolation of Colostrum Proteins

Lysis buffer (8 M urea (Sigma, St. Louis, MO, USA), 1% proteinase inhibitors (Calbiochem, Darmstadt, Germany) was added to samples, after which all samples were sonicated and centrifuged (12,000× *g*, 10 min, 4 °C). Supernatants were transferred to new centrifuge tubes, and protein concentrations were determined using a BCA kit (Solarbio, Beijing, China).

### 2.4. Protein In-Solution Digestion and Tagging

Proteins were hydrolyzed by trypsin using a kit (Promega, Madison, WI, USA) according to the manufacturer’s instructions, and digested peptides were tagged using labeling reagent from a TMT kit (Thermo, Waltham, MA, USA) labeling reagent.

### 2.5. Classification by High-Performance Liquid Chromatography (HPLC) and Analysis with Liquid Chromatography (LC) Mass Spectrometry (MS)

The labeled peptides were fractionated by HPLC at high pH, the chromatographic column was an Agilent 300 Extend C18 (5 μm particle size, 4.6 mm ID, 250 mm length). The operation was as follows: peptide-fractionation gradient was 8–32% acetonitrile, pH 9; 60 components were separated in 60 min and then combined into 9 components. All peptides were freeze-dried under a vacuum, and then separated by a NanoElute ultraperformance liquid chromatography (UPLC) system (Waters, Milford, MA, USA). Mobile phase A was 0.1% formic acid solution; mobile phase B was acetonitrile solution containing 0.1% formic acid. Liquid gradient parameters: 0–43 min, 6–22% B; 43–56 min, 22–30% B; 56–58 min, 30–80% B; 58–60, 80% B. Flow rate was maintained at 300 nL/min. Samples were injected into the capillary ion source of ionization and then analyzed using TOF Pro MS. The ion source voltage was 1.4 kV. The parent precursor ions and their secondary fragments were analyzed by TOF. The range of the secondary mass spectrum was 100–1700 m/z.

### 2.6. MS Data Analysis

Mass spectral data were searched using Maxquant (v1.6.5.0) software (http://www.maxquant.org/), with the following parameters: the database was UniProt_Sus scrofa, and digestion method was Trypsin/P with 2 missed cut sites. The first-order precursor ion mass error tolerance of the first search and main search were both 70 ppm, and the tolerance of the mass error of the secondary fragment ions was 0.04 Da. Fixed modifications were cysteine alkylation, variable modifications were methionine oxidation, and N-terminal acetylation of the protein. The false positive rate (FDR) for protein identification and the peptide-spectrum matches (PSM) identification was 1%.

### 2.7. Screening of Differentially Expressed Proteins

Quantitative values of specific peptides in samples were converted into log2 values. The PP and PC groups were compared with the CC group, and proteins with fold changes in expression of over 1.3 were considered differentially expressed when *p*-value was less than 0.05.

### 2.8. Bioinformatics Analysis

GO annotations were analyzed using InterProScan v.5.14-53.0 (http://www.ebi.ac.uk/interpro/), and KEGG annotations were analyzed using KAAS v.2.0 (http://www.genome.jp/kaas-bin/kaas_main).

### 2.9. Statistical Analysis

Microsoft Excel 2017 was used to process the data and make graphs; the two-sample two-tailed T test in SPSS19.0 (IBM, Armonk, NY, USA) was used to identify the significance of differential expression between two groups. A *p*-value < 0.05 was considered significant.

## 3. Results

### 3.1. Differentially Expressed Proteins

**Figure 1:**
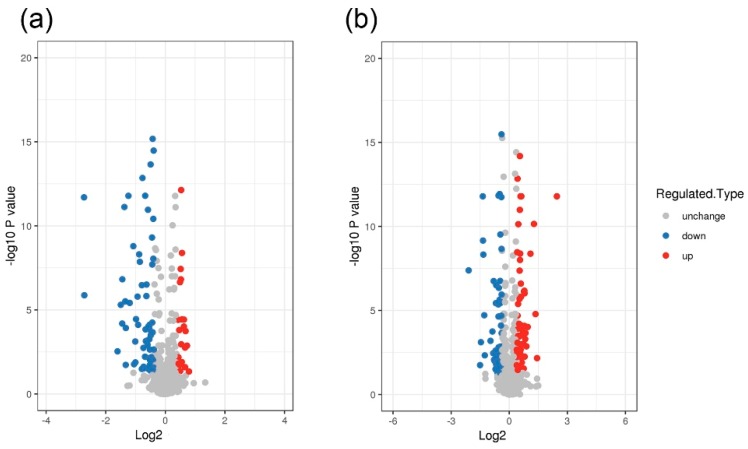
(**a**) Volcano plot of gestation-pen and farrowing-pen combined housing system (PP) vs gestation-crate and farrowing-crate combined housing system (CC) differential expression protein. (**b**) Volcano plot of gestation-pen and farrowing-crate combined housing system (PC) vs CC differential expression protein. Note: Ratio of differential protein expression over 1.3 was the significant change threshold (*p* < 0.05).

As shown in Figure 1a, 93 proteins with significant differences were detected in the comparison of PP versus CC (*p* < 0.05), including 24 upregulated proteins and 69 downregulated proteins. Additionally, as shown in Figure 1b, 126 proteins with significant differences were detected in the comparison of PC versus CC (*p* < 0.05), including 70 upregulated proteins and 56 downregulated proteins.

### 3.2. GO Functional Annotation

**Figure 2:**
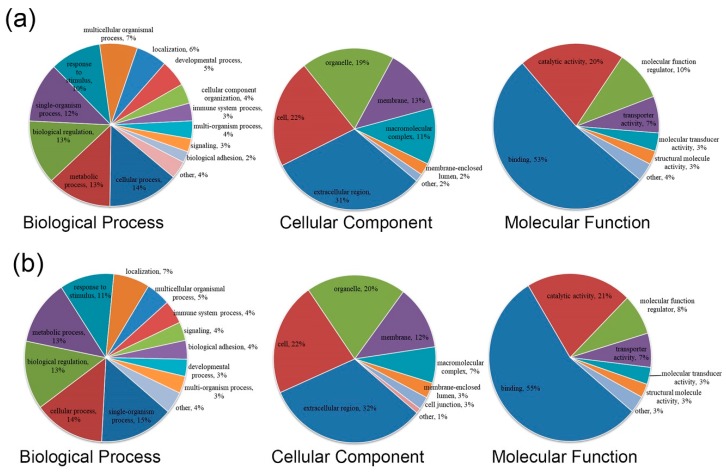
(**a**) Gene-ontology (GO) function annotation of PP vs. CC differential expression protein. (**b**) GO function annotation of PC vs. CC differential expression protein.

As shown in Figure 2a, GO functional annotations were obtained for the 93 differentially expressed proteins from the comparison of PP versus CC. Differentially expressed proteins were involved in biological processes, including cellular process (14%), metabolic process (13%), biological regulation (13%), and single-organism process (12%); cellular components, including extracellular region (31%), cell (22%), organelle (19%), and membrane (13%); and molecular functions, including binding (53%), catalytic activity (20%), and molecular function regulator (10%).

As shown in Figure 2b, differentially expressed proteins from the comparison of PC versus CC were involved in biological processes, including single-organism process (15%), cellular process (14%), biological regulation (13%), metabolic process (11%), and response to stimulus (11%); cellular components, including extracellular region (32%), cell (22%), organelle (20%), and membrane (12%); and molecular functions, including binding (55%) and catalytic activity (21%).

### 3.3. KEGG Pathway Enrichment of Differentially Expressed Proteins

**Figure 3:**
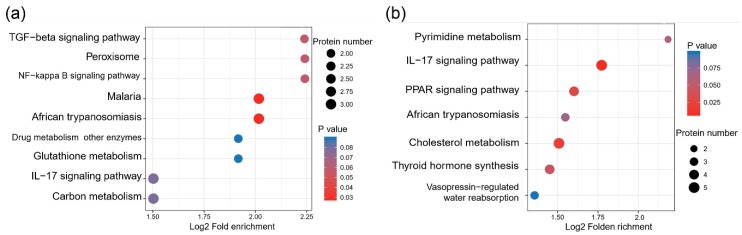
(**a**) Kyoto Encyclopedia of Genes and Genomes (KEGG) pathway enrichment of PP vs CC differential proteins. (**b**) KEGG pathway enrichment of PC vs. CC differential proteins. Note: Vertical axis of bubble chart is functional classification or pathway, enrichment test by Fisher’s exact test.

As shown in Figure 3a, differentially expressed proteins in the comparison of PP versus CC were enriched in the transforming growth factor (TGF)-β signaling pathway, peroxisome, nuclear factor (NF)-κ B signaling pathway, drug-metabolism enzymes, glutathione metabolism, interleukin (IL)-17 signaling pathway, carbon metabolism, African trypanosomiasis, and malaria, the latter two of which were significantly enriched (*p* < 0.05). As shown in Table 2, TGF-β3 was downregulated in the TGF-β signaling pathway, and differentially expressed proteins enriched in the IL-17 signaling pathway, including protein S100, calcium-binding protein, and heat-shock protein, were downregulated. All differentially expressed proteins enriched in the peroxisome and carbon metabolism pathways were also downregulated.

As shown in Figure 3b, the differentially expressed proteins in the comparison of PC versus CC were enriched in vasopressin-regulated water reabsorption, IL-17 signaling pathway, African trypanosomiasis, pyrimidine metabolism, cholesterol metabolism, thyroid-hormone synthesis, and peroxisome proliferator-activated receptor (PPAR) signaling pathway (*p* < 0.05). Additionally, as shown in Table 3, low-density lipoprotein receptor-related protein 2, lipoprotein lipase, and angiopoietin-related protein 4 were upregulated in the TGF-β signaling pathway, whereas apolipoprotein C-III and apolipoprotein A-I were downregulated; among the differentially expressed proteins in thyroid hormone synthesis, thyroxine-binding globulin was downregulated, whereas low-density lipoprotein receptor-related protein 2 was upregulated.

## 4. Discussion

Crates can cause chronic stress in sows, and high-cortisol concentrations were observed under chronic stress conditions [16]. Glucocorticoids, such as cortisol, are involved in the regulation of the immune system in the context of chronic stress [17,18]. In this study, the comparison of PP versus CC showed less significant differences in proteins than the comparison of PC versus CC. We speculate that this result may have been caused by housing changes, that is, transfer from pen to crate, resulting in more prenatal stress and activity restriction [16]. Yun et al. also demonstrated that nesting materials could cause changes in a sow’s metabolic status [6], and oxytocin and prolactin levels [7].

Studies have shown that chronic stress affects tumor necrosis factor-α, IL-1β, and IL-6 levels [19]. In the present study, differentially expressed proteins in the comparison of PP versus CC, and PC versus CC were mainly enriched in the IL-17 signaling pathway, indicating that the housing environment during pregnancy could affect the IL-17 signaling pathway. IL-17 is a potent proinflammatory cytokine [20] that is involved in inflammatory response, antifungal infection, and extracellular bacterial sensing. In the comparison of PP versus CC, the IL-17 signaling pathway was downregulated, which could be related to the induction of chronic inflammation under the conditions encountered in the CC group [21]; the conditions encountered in the PP group could alleviate this negative impact. As the main effector of T-helper 17 (Th17) cells, IL-17 is induced by TGF-β, IL-6, and IL-23 [22], and the combination of IL-17α with IL-17 receptor activates NF-κB [23]. These above effects were also observed in the comparison of PP versus CC; differentially expressed proteins were enriched in the TGF-β and NF-κB signaling pathways, and the TGF-β3 protein was downregulated, suggesting that PP caused less stress in sows. Notably, TGF-β plays important roles in gastrointestinal development of newborn piglets [24]: it is involved in mediating the differentiation of IgA, IgG, and IgM; and regulates B-cell responses to lipopolysaccharide and rotavirus [25]. Thus, downregulation of the TGF-β3 protein may be detrimental to the early growth and development of piglets.

Milk synthesis and secretion is regulated by hormones and nutritional metabolism [26,27]. GO annotations of proteins that were differentially expressed in the comparisons of PP versus CC, and of PC versus CC showed the involvement of metabolic processes. Moreover, in the comparison of PP versus CC, differentially expressed proteins were enriched in the glutathione pathway, and glutathione S-transferase was upregulated. Glutathione eliminates free radicals to reduce oxidative stress [28], and glutathione S-transferase catalyzes the production of reduced glutathione, which is conjugated with electron complexes to exert antioxidant effects. Taken together, these findings demonstrated that PP reduced oxidative stress in sows. Peroxisome, carbon metabolism, cholesterol metabolism, and the PPAR signaling pathway were involved in energy metabolism. Moreover, differentially expressed proteins in the comparison of PC versus CC were enriched in the PPAR signaling pathway and involved in fat metabolism. Indeed, the PPAR signaling pathway plays key roles in adipocyte differentiation, cholesterol metabolism, and fatty-acid metabolism [29,30]. In our association experiment, different colostrum lactose and fat contents were found between sows in different combined housing systems (e.g., lactose and fat content were, respectively, 11.55% and 7.52% in CC, and 12.02% and 5.18% in PP, unpublished), which supported our hypothesis of altered metabolic pathways. Peroxisomes are also involved in the metabolism of fatty acids and other lipids, and in the regulation of oxidative stress [31]. In our study, proteins involved in the peroxisome and carbon metabolism were downregulated in the comparison of PP versus CC. Thus, the housing environment affected fat metabolism in sows, and could thus alter the fat contents of sow milk, thereby affecting energy intake by newborn piglets.

## 5. Conclusions

In this study, we found that differentially expressed proteins in the comparisons of PC and PP groups with the CC group were mainly involved in the immune and metabolic pathways, particularly fat metabolism, indicating that the housing environment may affect the immune system and metabolic processes of sows. Additionally, PP may result in reduced stress in sows, as supported by the downregulation of the IL-17 signaling pathway and upregulation of glutathione S-transferase. Moreover, downregulated proteins related to the peroxisome and carbon metabolism in the comparison of PP versus CC may result in changes of fat content in sow milk, and proteins enriched in the PPAR signaling pathway in the comparison of PC versus CC may also be involved in fat metabolism. These findings provide important insights into the housing and management of sows to improve milk quality. However, the key proteins need to be determined to verify these hypotheses, and further studies are needed to find out the underlying mechanisms by which housing might affect piglets through dam milk.

## Figures and Tables

**Table 1:** Feeding standards during pregnancy and lactation.

Nutrient Composition	Pregnancy 0 to 90 Days	Pregnancy 90 to 107 Days	Pregnancy 107 Days to Weaning
DE (Mcal/kg)	3.0	3.2	3.39
CP (%)	14.66	15.47	18.31
SID Lys (%)	0.6	0.65	1.00

Diet-nutrient concentration was calculated from the nutrient composition of raw materials in the NRC (2012) database. DE, digestible energy; CP, crude protein; SID, standardized ileal digestibility; Lys, lysine.

**Table 2:** KEGG pathway enrichment of PP vs CC differential proteins.

KEGG Pathway	Protein Accession	Protein Description	Regulated Type
TGF-beta signaling pathway	F1S6B5	Fibromodulin	Up
TGF-beta signaling pathway	P15203	Transforming growth factor beta-3	Down
IL-17 signaling pathway	C3S7K5	Protein S100	Down
IL-17 signaling pathway	C3S7K6	Calcium-binding protein	Down
IL-17 signaling pathway	A0A287A9T4	Heat shock protein	Down
African trypanosomiasis	P01965	Hemoglobin subunit alpha	Down
African trypanosomiasis	A0A286ZJL9	Uncharacterized protein	Up
African trypanosomiasis	F1RII7	Hemoglobin subunit beta	Down
NF-kappa B signaling pathway	A0A286ZJL9	Uncharacterized protein	Up
NF-kappa B signaling pathway	A0A287B5Y6	Lipopolysaccharide-binding protein precursor	Down
Glutathione metabolism	F1RIF8	6-phosphogluconate dehydrogenase	Down
Glutathione metabolism	P80031	Glutathione S-transferase P	Up
Malaria	P01965	Hemoglobin subunit alpha	Down
Malaria	P15203	Transforming growth factor beta-3	Down
Malaria	F1RII7	Hemoglobin subunit beta	Down
Peroxisome	F1S3Y7	Xanthine dehydrogenase/oxidase	Down
Peroxisome	A0A287APD5	Long-chain-fatty-acid-CoA ligase 3	Down
Carbon metabolism	F1RIF8	6-phosphogluconate dehydrogenase	Down
Carbon metabolism	A0A287BBI5	Glyceraldehyde-3-phosphate dehydrogenase, decarboxylating	Down
Carbon metabolism	I3LK59	Enolase 1	Down
Drug metabolism other enzymes	F1S3Y7	Xanthine dehydrogenase	Down
Drug metabolism other enzymes	P80031	Glutathione S-transferase P	Up

**Table 3:** KEGG pathway enrichment of PC vs. CC differential proteins. Note: PPAR, peroxisome proliferator-activated receptor.

KEGG Pathway	Protein Accession	Protein Description	Regulated Type
Vasopressin-regulated water reabsorption	A0A287BNU5	RAB5B	Up
Vasopressin-regulated water reabsorption	F2Z536	Dynein light chain	Down
Vasopressin-regulated water reabsorption	A0A287BN36	Ras-related protein Rab-5C	Up
IL-17 signaling pathway	F1RRX1	Neutrophil gelatinase-associated lipocalin precursor	Down
IL-17 signaling pathway	C3S7K5	Protein S100	Down
IL-17 signaling pathway	C3S7K6	Calcium-binding protein A9	Down
IL-17 signaling pathway	A0A287A9T4	Heat shock protein	Up
IL-17 signaling pathway	Q29092	Endoplasmin	Up
African trypanosomiasis	P01965	Hemoglobin subunit alpha	Down
African trypanosomiasis	K7GM40	Apolipoprotein A-I	Down
African trypanosomiasis	F1RII7	Hemoglobin subunit beta	Down
Pyrimidine metabolism	Q2EN76	Nucleoside diphosphate kinase B	Up
Pyrimidine metabolism	I3LH72	Ectonucleoside triphosphate Diphosphohydrolase 6	Down
Cholesterol metabolism	P27917	Apolipoprotein C-III	Down
Cholesterol metabolism	A0A287AZ36	Low-density lipoprotein receptor-related protein 2	Up
Cholesterol metabolism	P49923	Lipoprotein lipase	Up
Cholesterol metabolism	K7GM40	Apolipoprotein A-I	Down
Cholesterol metabolism	Q2TNK5	Angiopoietin-related protein 4	Up
Thyroid hormone synthesis	P50390	Transthyretin	Down
Thyroid hormone synthesis	F1RXM6	Thyroxine-binding globulin	Down
Thyroid hormone synthesis	A0A287AZ36	Low-density lipoprotein receptor-related protein 2	Up
Thyroid hormone synthesis	Q29092	Endoplasmin	Up
PPAR signaling pathway	P27917	Apolipoprotein C-III	Down
PPAR signaling pathway	P49923	Lipoprotein lipase	Up
PPAR signaling pathway	K7GM40	Apolipoprotein A-I	Down
PPAR signaling pathway	Q2TNK5	Angiopoietin-related protein 4	Up
